# Mental Health Risk and Protective Factors of Nigerian Male Asylum Seekers Hosted in Southern Italy: a Culturally Sensitive Quantitative Investigation

**DOI:** 10.1007/s40615-022-01260-3

**Published:** 2022-02-15

**Authors:** Francesca Tessitore, Anna Parola, Giorgia Margherita

**Affiliations:** 1 Department of Humanities, Philosophy and Education, University of Salerno, Fisciano, Italy; 2 Department of Humanities, University of Naples Federico II, Naples, Italy

**Keywords:** Clinical assessment, Trauma, Resilience, Post-migratory difficulties, Nigerian asylum seekers, Italy

## Abstract

This study provides a culturally sensitive quantitative investigation aimed at assessing the post-traumatic symptomatology, post-migratory difficulties, and resilience of 36 Nigerian male asylum seekers hosted in the province of Caserta, South Italy. A survey composed by the Harvard Trauma Questionnaire-Revised (HTQ-R), the Post-Migratory Checklist (PLMD), and the Connor-Davidson Resilience Scale (CD-RISC) was administered to participants. Descriptive and correlation analyses were made in order to describe the mental health risk and protective factors and understand the relation between these. A linear regression analysis was used to evaluate the influence of post-migratory difficulties and resilience on PTSD. Stratified bivariate analyses were also computed to detect PTSD group and no-PTSD group differences about post-migration difficulties and resilience levels. Regression analysis showed that PMLD numbers significantly increased the risk of having PTSD. No significant effect emerged for the level of resilience. Statistically significant differences between the PTSD group and non-PTSD group in relation to post-migratory difficulties were also found. No differences in the resilience factors emerged. The results offer a glimpse into a specific ethnic group of asylum seekers and its mental health risks and protective factors, taking into consideration the specificities of their past and current life-story experiences. Clinical implications for professionals working in the field of forced migration will be outlined.

## Introduction

In the last few years, the field of research on the mental health status of forcibly displaced people has largely demonstrated a high risk for asylum seekers, compared to refugees and voluntary migrants, to develop psychological and psychopathological disturbances, such as post-traumatic stress disorders, anxiety, and depression, with high rates of comorbidity, as well as high rates of suicide and self-harm [25, 47].

Major risk factors for mental health concerns not only the experiences of extreme traumatization that asylum seekers live before and during the journey but also the large variety of post-migration stressors and uncertainties that they live once arrived in the context of reception: the lack of opportunity to work or study due to their asylum status, uncertainties connected to the legal procedures to achieve asylum, and acculturation challenges [33, 56, 58, 57]. In this sense, the trauma lived by asylum seekers revealed a clear and structural multidimensional and multi-temporal character [36].

Research on protective factors for mental health has also demonstrated that despite devastating experiences, most refugees are extremely resilient [2, 4, 28, 50] even though there is no accordance in the conceptualization of resilience, which widely ranges from a personality trait that enable one to thrive in the face of adversity [13] to an unfolding process which includes individual as well as community and cultural elements [61]. Overall, a wide range of variables seem to contribute to the development of great levels of resilience: individual qualities and strengths, family, social and community support, and meaningful relationships [11].

Despite the field of research on the mental health of displaced people being very wide and still ongoing, it is undeniable that the experiences of asylum seekers might differ profoundly from one another. To a large degree, previous research has tended not to differentiate between them. This tendency to homogenize, even though it has allowed researchers to carry out wide investigations on great numbers of asylum seekers, has not enriched professional’s understanding of the different ways in which asylum seekers can live the adversities, from their ethnicity, cultural belonging, and the specificities of their context of origin to journey experiences and context of reception. According to Solberg et al. [53], this has produced several obstacles against the development of a situated knowledge on asylum seekers’ characteristics and a limited understanding of the complex nature of the trauma they live. Thus, the need to carry out culturally sensitive studies within the context of quantitative investigations around the issue of the mental health of asylum seekers appears even more urgent.

## Nigerian Migrants Towards Italy

In the last few years, Italy has been one of the top countries in Europe in terms of the number of non-voluntary migrant arrivals. Even though the nationality of migrants entering Italy has varied significantly over the years, since 2015, the largest group of non-voluntary migrants who has requested international protection in Italy has come from West Africa. According to the latest data of the Italian Ministry of Interior [38], since 2015, Nigerian men have made up the majority of asylum seekers in the Italian territory. Most of them are young men, between the age of 20 and 35, who arrived in Italy through the main migratory route of the Central Mediterranean Sea from Libya.

As largely documented, the journey from Nigeria to Italy is characterized by a specific constellation of extreme traumatic events. First, the crossing of the Sahara Desert, which often lasts several days or weeks, and is characterized by starvation, dehydration, and endured violence. Second, the experience of imprisonment in Libyan detention camps where most Nigerian migrants are forced to work and tortured. Finally, the crossing of the Mediterranean Sea, with its heavy load in terms of survival: the UNHCR estimated that, from 2015 to 2018, around 15,000 migrants died in the Mediterranean Sea.

In the case of Nigerian people, all these experiences often add onto existing pre-migratory experiences of political or religious persecutions by terroristic or cult groups, disadvantaged familiar, social, and economic conditions, violence, and corruptions. These conditions often represent the main reasons why Nigerians flee from their country. In the case of women, it is estimated that around 80% of them are trafficked [29].

To the best of the authors knowledge, despite the high numbers of Nigerians in Europe and Italy, there has been very little research specifically focused on this ethnicity. Broadly, Smigelsky et al. [51] carried out a situated investigation on sub-Saharan Africans’ mental health conditions, reporting high levels of post-traumatic symptomatology. However, where research has specifically investigated Nigerian migrants’ characteristics or experiences, they have mainly focused on the experiences of women and, in particular, on those who have survived trafficking [22].

## Objectives

The current study is part of a wider research project aimed to assess the mental health status and explore the subjective meanings around the migratory experiences of Nigerian asylum seekers hosted in Italy in-depth [54–56]. The whole research project was shaped by a mixed methodology which combined quantitative (i.e., questionnaires aimed at investigating risks and protective factors for mental health) and qualitative investigations (i.e., in-depth interviews aimed at exploring the subjective meanings attributed to the pre-, peri-, and post-migratory experiences). Here, the results of the quantitative mental health assessment carried out on a group of 36 Nigerian male asylum seekers hosted in some Extraordinary Reception Centers located in the Campania Region, Southern Italy, are presented. Since the present study is the first specifically focused on a sample of Nigerian male asylum seekers, it predominantley had a descriptive and explorative nature. The general aim was to assess the post-traumatic symptomatology, perceived post-migratory difficulties, and resilience levels of Nigerian male asylum seekers, and to investigate the relationship between all these variables, along with the effects of post-migratory difficulties and resilience on PTSD. Furthermore, socio-demographic variables, post-migratory difficulties, and resilience were also investigated according to the presence or absence of PTSD. These variables were chosen following the constructs widely used in literature for the investigation of mental health risks and protective factors. The authors expected the following: (a) resilience to be negatively associated with PTSD, (b) post-migration difficulties to be positively associated with PTSD, and (c) higher levels of post-migration difficulties and lower levels of resilience in the PTSD group compared to the non-PTSD group.

By avoiding the generalization of the results obtained to the whole category of Nigerian male asylum seekers hosted in Italy, the authors believe that the present investigation might offer a glimpse into a specific and still unexplored group of asylum seekers, taking into consideration the specificities of their ethnicity as well as their past and current conditions, and enriching the scientific literature on asylum seekers’ mental health status.

## Materials and Method

### The Context and the Socio-political Climate of the Study

This study was carried out in 2019 in some Extraordinary Reception Centers (Italian acronym: CAS) located in the Campania Region and managed by different private cooperatives. The CAS’s were born in 2015 as a consequence of the so-called North-African Emergency, during which thousands of migrants arrived in Italy from North Africa across the main route of the Central Mediterranean Sea from Libya. At that time, due to a lack of available places in the “regular” shelters (the SAI system — ex SIPROIMI/SPRAR) active until that moment, the Italian authorities created the parallel system of the CAS’s. The CAS’s are still overseen by the Ministry of Interior provincial offices which allocate funds to private or third sector providers. The Extraordinary Reception Centers were born as emergency and provisional solution, but they rapidly ended up being the most common reception centers for asylum seekers. In accordance with the initial dispositions of the Ministry of Interior, these centers granted asylum seekers with a wide range of services (i.e., board and lodging, teaching of the Italian language, medical and psychological support, and legal advice).

Since 2018, several political and social changes have been registered in Italy around the issue of non-voluntary migrants’ reception. To a large degree, these changes have been the consequence of a live political propaganda created by Italian right-wing parties which not only lead to some concrete changes in the Italian system of primary and secondary reception but also produced a distorted representation of the migratory phenomenon, nourishing the image of the migrants as “enemies to combat” [56]. As a consequence, at the time of the study, an increasingly intolerant atmosphere towards immigrants had started to spread throughout Italy. In particular, in the South of Italy where the social and economic circumstances are more disadvantaged than the North, several demonstrations of local communities against municipalities and cooperatives engaged in non-voluntary migrants’ reception were registered. Most of these demonstrations happened in the Campania Region which was the top region in the South of Italy and the second throughout Italy in terms of the number of primary reception centers [26]. At the end of 2018, some great changes within the Italian reception system were endorsed by the approval of the so-called Security Decrees (Law no. 132 of 1 December 2018; Law n. 77 of 8 August 2019). Among other changes, the first “Security Decree” established the CAS’s as the official primary reception centers but with significant cuts to resources and services which were previously granted to asylum seekers, such as the teaching of the Italian language and the psychological support. Furthermore, big centers able to host a high number of asylum seekers were preferred to small centers in which beneficiaries could have been better taken care of and followed in their integration paths. The most common form of protection granted to asylum seekers in Italy, the humanitarian protection, was also abolished.

Recently, new and better adjustments were applied in Italy in order to correct some of the amendments of the “Security Decrees” which were declared unconstitutional. To date, the Decrees have been replaced by the so-called Immigration Decrees, promoted by the new Ministry of Interior Luciana Lamorgese.

### Participants Selection

According to the purposes of the study, participants were selected on the basis of the following selection criteria:

- Being Nigerian male asylum seekers, according to the definition of the UNHCR;
- Being able to write and read in English.

Before proceeding with the data collection, the asylum seekers respecting the selection criteria were met collectively at each center, informed of the aims of the study, and asked to express interest in participating. During the first collective meeting, the researcher who carried out the administration (the first author) carefully explained the research aims and procedures, as well as the role assumed by the psychologist/researcher during the research.

Out of a total of 45 Nigerians’ asylum seekers met, 9 declared themselves not available to take part in the study. A total of 36 participants took part in the research: 12 in the first center, 15 in the second, and 9 in the third.

### Setting and Procedures

All the research procedures were performed in a room made available by the centers, at the presence of the participant, the researcher, and a Nigerian cultural consultant who had already worked with the participant during the several center’s activities and helped the researcher with any specific language issues.

After the first collective meeting, a total of two individual meetings with each participant were performed. In the first meeting, participants signed the consent form to take part in the research and completed a form with social-demographic information. In a second meeting, a survey was administered in order to collect information regarding post-traumatic symptom levels, post-migratory difficulties, and resilient characteristics.

All the meetings were performed in English.

### Instruments

A social-demographic schedule was developed ad hoc in order to collect some information about participants. Specifically, the schedule was composed by three sections:

- *Personal information*: personal information about region of provenance, age, sex, education level, religion, marriage, children, and studying or working activities in the motherland were collected.
- *Migratory information*: participants were asked to write freely the principal reasons why they fled from their motherland.
- *Post-migratory information*: information about some post-migratory determinants (i.e., year of arrival in Italy, status of the asylum claim, and presence or absence of work) were collected.

The survey for the assessment of risk and protective factors for mental health was composed by the following instrument:

- *The Harvard Trauma Questionnaire-Revised* (HTQ-R) [39, 40]. The HTQ is a self-report questionnaire composed of different parts aimed to assess trauma events; personal description of trauma events; presence of brain injuries; and presence and qualities of post-traumatic symptoms. For the purposes of the present study, only the first 16 items of part IV of the HTQ-R were administrated in order to measure the post-traumatic symptomatology. Participants were asked to describe how much some experiences bothered them during the last week on a 4-point Likert scale from 1 (not at all) to 4 (extremely). Part IV of the HTQ-R was derived from the *Diagnostic and Statistical Manual of Mental Disorders*, *Third Edition-Revised* (DSM-III-R) and later the *Diagnostic and Statistical Manual, Fourth Edition* (DSM-IV) criteria for PTSD using three sub-domains: re-experiencing traumatic events (4 items), avoidance and numbing (7 items), and psychological arousal (5 items). A DSM-IV score can be obtained by adding up the scores of the first 16 items answered and dividing by the total number of the answered items. Individuals with scores on DSM-IV and/or total ≥ 2.5 could be considered symptomatic for PTSD. The HTQ has been shown to have high internal consistency in African refugees [41]. For this study, Cronbach’s alpha for the three subscales were 0.72, 0.75, and 0.63.
- *The Post-migration Living Difficulties Scale* (PMLD) [49]. The PMLD is a self-report checklist aimed to assess the levels of stress due to typical post-migration stressors. It consists of a list of 21 possible post-migration living difficulties. Respondents were asked to indicate the extend to which they were troubled by any of the mentioned living problems, ranging on a 5-point scale from “no problem at all” to “very serious problem.” The checklist was shortened to 12 items for the purpose of the present study with the aim of assessing difficulties in the following areas: communication; discrimination; separation from family; worry about family back at home; being unable to return home in an emergency; not being able to find work; being fearful of being sent back to the country of origin in the future; worries about access to treatment for health problems, loneliness, and boredom; isolation; experience of perceived tolerance; and thoughts regarding the right or wrong decision to come in Italy. As highlighted by Silove et al. [49], each item should be treated as a separate incident of stressor.
- *The Connor-Davidson Resilience Scale* (CD-RISC) [13]. The CD-RISC is a self-report measure comprising 25 items rated on a 5-point Likert scale from 0 (not at all true) to 4 (true nearly all of the time). The CD-RISC yields a total resilience score ranging from 0 to 100, with higher scores reflecting greater resilience. As highlighted by Connor and Davidson [13], factor analysis has demonstrated a five-factor structure: factor 1 relates to personal competence, tenacity, and high standards; factor 2 relates to trust in one’s instincts, tolerance of negative affects, and resolution in the face of stress; factor 3 relates to the acceptance of changes and feelings of security in relationships; factor 4 relates to perceived control; and factor 5 relates to spiritual beliefs. Despite the CD-RISC not having been developed specifically on refugees, it had been used in various cross-cultural studies [6, 62] as well as assessed on refugees [12]. For this study, Cronbach’s alpha for the five factors were 0.88 for factor 1—personal competence, 0.68 for factor 2—tolerance of negative, 0.68 for factor 3—positive acceptance of changes, 0.55 for factor 4—perceived control, and 0.61 for factor 5—spiritual influence.

### Data Analysis

Descriptive statistics (mean and standard deviation) were used to study the prevalence of post-traumatic symptomatology, post-migration difficulties, and resilience levels.

Then, correlations between variables were performed to study the relationship between resilience level, PMLD, and PTSD. A linear regression analysis was used to evaluate the influence of the number of PLMD (the number of serious or very serious problems) on the likelihood of PTSD. The resilience level was also inserted in the model.

Finally, stratified bivariate analyses by presence/absence of PTSD were also computed. The presence of PTSD refers to individuals with scores of ≥ 2.5. Student’s *t*-tests and bootstrap analysis (95% CI) estimated with 1000 bootstrap samples were performed to detect PTSD group and no-PTSD group differences about post-migration difficulties and resilience levels. The effects of the differences were evaluated with Cohen’s *d*. Student’s *t*-tests and *χ*^2^ tests were used to analyze differences in socio-demographic variables between the PTSD group and no-PTSD group according to the type of variables (age, number of years of education, mean time of arrival in Italy, married, son/daughter, religion, working in country, studying in country, and reason why fled from country).

Analyses were performed through the Statistical Package for Social Sciences (IBM SPSS Statistics 24.0).

## Results

### Participants’ Characteristics

As shown in Table 1, participants had a mean age of 27.47 (SD = 7.28). All of them were Nigerians. Specifically, 26 came from Edo State, 5 from Delta State, 1 from Ebony State, 1 from Lagos, 1 from Benue State, 1 from Enugu State, and 1 from Ondo State.

**Table 1:** Characteristics of participants

*Total of participants* (*n* = 36)
Personal information
*Age*: 27.47 (SD = 7.28) *Gender*: Male *Country of origin*: Nigeria *Number of years of education*: 11.7 (SD = 3.78) *Religion*: 55.6% Christian; 44.4% Muslim *Married*: 80.6% no; 13.9% yes; 5.6% divorced *Children*: 80.6% no; 19.4% yes *Working in country of origin*: 69.4% *Studying in country*: 16.7%
Migratory information
Reasons why they fled from the country^1^ *Community/familiar threat*: 30.6% *Improvement living condition*: 27.8% *Political persecution*: 13.9% *Terroristic persecution*: 11.1% *Escape from justice*: 8.3% *Religious persecution*: 5.6% *Gender persecution*: 2.8%
Post-migratory information
*Months in Italy*: 16.7% (SD = 8.05) *Evaluation of international protection request*: 94.4% no; 5.6% yes *Work in Italy*: 11.1% yes; 69.4% never; 19.4% sometimes

^1^The reasons why participants fled from country were categorized as follows:

- Community/familiar threat;

- Improvement of living conditions;

- Political persecution;

- Terroristic/religious persecution;

- Justice problems;

- Gender persecution

80.6% of participants were not married, and 80.6% had no children.

The majority of participants referred to fled from community/familiar threat (30.6%) and for improving their living conditions (27.9%). The rest of them fled from terroristic/religious persecution (17.5%), political persecution (13.9%), justice (8.3%), and gender persecution (2.8%).

Regarding their post-migratory conditions, despite participants having already been in Italy for 16.7 months, 94.4% of them were still waiting for the evaluation of their international requests, while 5.6% had already done the evaluation and were waiting for the response.

### Post-traumatic Symptomatology

As shown in Table 2, considering all participants, the mean value for the DSM-IV PTSD score of the HTQ-R [39] did not pass the cut-off value (≥ 2.5).

**Table 2:** Traumatic levels HTQ-R

Sub-domains	M	SD	*N*
Re-experiencing traumatic events	2.39	0.82	36
Avoidance and numbing	2.31	0.71	36
Psychological arousal	2.15	0.73	36
**DSM-IV PTSD**	**2.28**	0.63	36
**DSM-IV PTSD ≥ 2.5**	**2.93**	0.62	14

*Note*: *M*, mean; *SD*, standard deviation

Considering each sub-domain in isolation, higher scores were registered in the sub-domains “re-experiencing traumatic events” and “avoidance and numbing” that emerged as the most common modalities to deal with the traumatic experiences that participants’ lived.

Looking at individual participants, 14 out to 36 participants reported values higher than the cut-off for the DSM-IV score resulting symptomatic for PTSD.

### Post-migratory Difficulties

In line with Silove et al. [49], each item of the PMLD was considered as separated. As shown in Table 3, participants reported higher levels of difficulty in many areas. In particular, high difficulty levels emerged in relation to “worry about family at home,” “not being able to find work,” “being fearful of being sent back to their country of origin,” and “worries about not getting access to treatment of health problems.”

**Table 3:** Post-migratory difficulties (PMLD) (means and standard deviation, *n* = 36)

Items	M	SD
Communication difficulties	2.78	1.40
Discrimination	2.42	1.42
Separation from your family	2.75	1.61
Worry about family back at home	3.39	1.68
Being unable to return home in an emergency	2.94	1.82
Not being able to find work	3.39	1.42
Being fearful of being sent back to your country of origin in the future		
Worries about not getting access to treatment to health problems	3.22	1.66
Loneliness and boredom	3.22	1.66
Isolation	2.71	1.60
Satisfaction in Italy	2.47	1.48
Perceived level of tolerance in Italy	2.31	1.37
Fairness of decision to come to Italy	2.11	0.75

*Note*: *M*, mean; *SD*, standard deviation

44.4% of participants reported uncertainties regarding the degree of perceived tolerance displayed by Italians towards people of other races, cultures, and countries. Nevertheless, the majority of participants thought that the decision to come in Italy was the right one.

### Resilience Levels

As shown in Table 4, participants presented a mean value of resilience of 63.31, demonstrating moderately high values of resilience considering the score range 0–100.

**Table 4:** Resilient characteristics (CD-RISC)

**Factor**	M	SD	Min	Max
Factor 1 — personal competence	**23.36**	7.20	6	32
Factor 2 — tolerance of negative effects	**14.56**	5.32	2	26
Factor 3 — positive acceptance of changes	11.61	4.39	1	20
Factor 4 — perceived control	7.44	3.11	0	12
Factor 5 — spiritual influence	6.33	1.77	1	8
**Resilience total score**	**63.31**	18.06	14	92

*Note*: *M*, mean; *SD*, standard deviation

Higher values were reported in factor 1 which refers to the self-perception of personal competence as well as in factor 2 which refers to the tolerance of the negative effects of events.

### Relationship Between PTSD, PMLD, and CD-RISC

Correlation analysis (Table 5) showed positive associations between PTSD score and some of the PMLD dimensions: communication difficulties, discrimination, worry about family at home, loneliness and boredom, and isolation. No relationship emerged between PTSD score and resilience. Only a positive relationship emerged between level of resilience and the levels of satisfaction in Italy.

Regression analysis showed that PMLD numbers significantly increased the risk of having PTSD. No significant effect emerged for the level of resilience (Tables 5 and 6).

**Table 5:** Correlational analyses

	1	2	3	4	5	6	7	8	9	10	11	12	13	14	15
1. Resilience level	-														
2. Communication difficulties	− 0.006	-													
3. Discrimination	0.104	0.221	-												
4. Separation from your family	0.287	0.369*	0.396*	-											
5. Worry about family back at home	0.064	0.343*	0.373*	0.650**	-										
6. Being unable to return home in an emergency	− 0.227	0.299	− 0.068	0.327	0.587**	-									
7. Not being able to find work	0.011	0.102	0.229	0.469**	0.510**	0.385*	-								
8. Being fearful of being sent back to your country of origin in the future	0.151	− 0.027	0.208	0.278	0.266	0.485**	0.439**	-							
9. Worries about not getting access to treatment to health problems	− 0.057	0.034	0.420*	0.471**	0.389*	0.203	0.642**	0.425**	-						
10. Loneliness and boredom	0.211	0.163	0.549**	0.415*	0.497**	0.313	0.474**	0.526**	0.597**	-					
11. Isolation	0.16	0.025	0.554**	0.314	0.498**	0.179	0.222	0.386*	0.328	0.753**	-				
12. Satisfaction in Italy	0.373*	− 0.053	0.3	0.269	0.27	− 0.05	0.143	− 0.048	0.322	0.408*	0.363*	-			
13. Level of tolerance in Italy	− 0.163	0.161	0.197	0.047	0.147	0.068	0.039	0.107	0.279	0.028	− 0.023	0.022	-		
14. Fairness of decision to come to Italy	0.005	− 0.01	0.196	0.247	0.340*	0.058	0.178	− 0.082	0.272	0.264	0.358*	0.155	− 0.071	-	
15. PTSD Score	0.036	0.434**	0.474**	0.283	0.382*	0.271	0.011	0.139	0.175	0.362*	0.380*	0.228	0.172	− 0.009	-

*Note*. *p ≤ 0.05; **p ≤ 0.01

**Table 6:** Regression model

	*β*	SE	*t*	*p*	95% CI
Number of PMLD	0.44	0.034	2.846	0.008	[0.027, 0.164]
Resilience level	0.23	0.019	0.885	− 0.035	[− 0.035, 0.041]

*R*^2^ = 0.44

### PTSD Group and No-PTSD Group Analysis

Student’s *t*-tests are reported in Table 7. Statistically significant differences between the PTSD group and no-PTSD group were found. The PTSD group reported a higher mean than the no-PTSD for PMLD dimensions of discrimination, loneliness and boredom, and isolation. No differences in the resilience factors were found.

**Table 7:** Descriptive analyses, *t*-test, and effect sizes for the PTSD group and no-PTDS group

	No PTSD	PTSD	*T*_*(df)*_	*p*	95% CI	*d*
	M (SD) [95% CI]	M (SD) [95% CI]				
Personal competence	22.82 (7.75) [19.50, 25.85]	24.21 (6.43) [20.55, 27.43]	− 0.742_(34)_	0.463	[− 0.789, 0.439]	0.26
Tolerance of negative effects	14.54 (5.08) [12.38, 16.71]	14.57 (5.89) [11.17, 17.66]	0.063_(34)_	0.950	[− 0.444, 0.662]	0.02
Positive acceptance of changes	11.86 (4.47) [10.09, 13.58]	11.21 (4.39) [8.86, 13.43]	0.428_(34)_	0.672	[− 0.549, 0.690]	0.15
Perceived control	7.13 (3.31) [5.86, 8.47]	7.93 (2.81) [6.25, 9.25]	− 0.582_(34)_	0.564	[− 0.822, 0.591]	0.20
Spiritual influence	6.45 (1.62) [5.74, 7.09]	6.14 (2.03) [5.07, 7.12]	0.509_(34)_	0.614	[− 0.501, 0.805]	0.17
Resilience total score	62.81 (19.01) [54.56, 70.52]	64.07 (.17.14) [53.46, 72.07]	− 0.196_(34)_	0.846	[− 0.530, 0.513]	0.07
Communication difficulties	2.55 (1.44) [2.00, 3.19]	3.14 (1.29) [2.33, 3.79]	− 1.262_(34)_	0.215	[− 1.479, 0.390]	0.43
Discrimination	2.00 (1.41) [1.42, 2.63]	3.07 (1.21) [2.40, 3.80]	− 2.341_(34)_	**0.025**	[− 1.976, − 0.149]	0.81
Separation from your family	2.64 (1.62) [2.04, 3.32]	2.93 (1.64) [1.91, 3.64]	− 0.527_(34)_	0.603	[− 1.240, 0.964]	0.18
Worry about family back at home	3.09 (1.74) [2.38, 3.80]	3.86 (1.51) [2.86, 4.55]	− 1.351_(34)_	0.186	[− 1.714, 0.464]	0.47
Being unable to return home in an emergency	2.77 (1.87) [2.00, 3.62]	3.21 (1.76) [2.06, 4.00]	− 0.705_(34)_	0.486	[− 1.519, 0.972]	0.24
Not being able to find work	3.50 (1.44) [2.94, 4.10]	3.21 (1.42) [2.27, 3.80]	0.583_(34)_	0.564	[− 0.511, 1.476]	0.20
Being fearful of being sent back to your country of origin in the future	3.59 (1.71) [2.86, 4.27]	3.64 (1.55) [2.67, 4.31]	− 0.092_(34)_	0.927	[− 0.967, 1.166]	0.03
Worries about not getting access to treatment to health problems	3.09 (1.57) [2.45, 3.77]	3.43 (1.83) [2.30, 4.30]	− 0.590_(34)_	0.559	[− 1.337, 1.089]	0.20
Loneliness and boredom	2.27 (1.52) [1.65, 2.93]	3.46 (1.51) [2.60, 4,29]	− 2.245_(34)_	**0.032**	[− 2.250, − 0.049]	0.78
Isolation	2.00 (1.31) [1.50, 2.61]	3.21 (1.48) [2.40, 4.09]	− 2.582_(34)_	**0.014**	[− 2.258, − 0.238]	0.86
Satisfaction in Italy	2.00 (1.13) [1.58, 2.45]	2.79 (1.63) [2.08, 3.83]	− 1.725_(34)_	0.094	[− 1.891, 0.027]	0.56
Perceived level of tolerance in Italy	2.09 (0.75) [1.79, 2.41]	2.14 (0.77) [1.71, 2.55]	− 0.200_(34)_	0.842	[− 0.583, 0.470]	0.06
Fairness of decision to come to Italy	1.36 (0.73) [1.10, 1.69]	1.29 (0.73) [1.00, 1.73]	0.314_(34)_	0.756	[− 0.436, 0.524]	0.09

*Note*: *M*, mean; *SD*, standard deviation

Additional explorative analyses were conducted in an attempt to understand if traumatic dimensions might have related with some socio-demographical information. Statistically significant differences between the PTSD group and no-PTSD group emerged for religion. In the PTSD group, more individuals of Muslim religion were present (*χ*^2^_(1)_ = 6.756, *p* = 0.009).

No other associations emerged from the analysis probably due to the small sample size and the fact that the group of participants was highly homogeneous and very few differences emerged between individual personal characteristics.

## Discussion

The present study investigated mental health risk and protective factors in 36 Nigerian male asylum seekers hosted in some Extraordinary Reception Centers in the province of Caserta, Southern Italy, in an attempt to carry out a culturally sensitive investigation able to shed light on the specificities of a specific ethnic group and its mental health status.

Focusing on an often “ignored” target population, when compared with the female counterpart, this study offered the first assessment carried out specifically on Nigerian male asylum seekers both in Italy and worldwide.

First, social-demographic information revealed that most of participants (26 out of 36) came from Edo State which is known for being one of the cruelest states within the Niger Delta. Here, in addition to human trafficking which mainly involves women, poverty, criminality, corruption, violence, conflicts between bands, political, cult groups, and communities also contribute to the current mass migration [10]. According to participants, the main reasons why they fled from Nigeria regarded community/familiar threat, the possibility to improve their living conditions, and terroristic/religious persecution. Most of them referred to conflicts within the familiar context mainly inherent from heritage issues which ended up into persecution and threats. As shown in a previous study [55], the deep anchoring of Nigerian male asylum seekers’ departure to the vulnerable socio-political structure of Nigerian society produced a multitude of specific feelings that range from guilt, regret, and powerlessness that acquired the sense of failure of male supremacy usually promoted and supported in Nigerian cultures. This information could enlighten practitioners on the need to take into account the precise social and political nature of violence with the peculiar consequences it has on the individual and relational words of Nigerian men.

In order to evaluate the level of post-traumatic symptomatology, the first 16 items of the PART IV of the Harvard Trauma Questionnaire-Revised were administered. The other sections of the instrument, which are aimed at evaluating the specific types of traumatic events lived by refugees, were excluded due to the concerns these raised about the eventual negative consequences which the reading and need to recognize and check a list of devastating events could have had on participants. From the author’s point of view, these considerations should stimulate a wider debate about the ethical and cultural appropriateness of the methodologies used within the transcultural research and clinical practice as well as the need to develop more efficient instruments and methodologies able to dialogue with the cultural otherness [46, 55, 57, 59].

Overall, despite the devastating events lived by participants, most of them, 22 out of 36, resulted non-symptomatic to PTSD, confirming the need to avoid making a direct and causal link between the impact of the devastating events lived by asylum seekers and the development of post-traumatic syndromes. Broadly, this result stressed the need to consider the post-traumatic symptomatology as a construct able to give some important information about individual reactions to traumatic experiences but, at the same time, the need to go beyond it, in an attempt to explore and take into consideration the different levels (individual, relational, and contextual as well) involved in the migratory trauma [5, 21, 35, 36].

Regarding the post-migratory difficulties, participants reported higher levels of difficulties in relation to the following dimensions: “worry about family at home,” “not being able to find a work,” “being fearful of being sent back to their country of origin,” and “worries about not getting access to treatment of health problems.” Considering the specific socio-political and cultural nature of the post-migratory stressors, these worries might be interpreted as a result of the vulnerabilities introduced by the approval of the “Security Decrees” and the diffusion of a politic of fear [64], to the detriment of a politic of hospitality.

Regarding protective factors for mental health, in line with previous studies on refugees with different nationalities [4, 28], the results showed that Nigerian asylum seekers present moderately high levels of resilience, with higher values in the perception of personal competence and tolerance of negative effects. Higher levels of resilience seemed to correlate with greater levels of satisfaction towards Italy expressed by participants.

Regarding the correlation analyses, the positive associations between traumatic dimensions and most of the post-migratory difficulties explored were confirmed. Regression analysis also showed that the increase in post-migratory difficulties significantly influenced the risk of having PTSD. Broadly, our results were in line with a wide range of international studies which indicated the post-migratory and contextual determinants as the strongest indicators of mental health disorders [1, 7, 34, 42, 53]. In contrast with our assumption and with previous studies carried out on the general population [3, 37], no relationship emerged between PTSD and resilience. This result is, instead, in line with a previous investigation carried out by Arnetz et al. [2] which did not find a correlation between these constructs in refugees. The authors [2] explained the result highlighting the inadequacy of the instruments aimed to assess resilience on refugees. Accordingly, it might be important to highlight that the CD-RISC used in this study is mainly focused on person-centered variables and, despite it also having been used on refugee samples [12, 44], it might define resilience from a Western perspective which could be more appropriate to individualist cultures. In this sense, it should be taken into consideration that, in the wake of trauma, an individual from a collectivist culture may conceptualize resilience in terms of community more than in terms of self [9]. This evidence is also supported by the low values of Cronbach’s alpha in the CD-RISC measure.

Finally, the analysis of the differences according to the presence or absence of PTSD, as expected, showed higher levels of discrimination, loneliness and boredom, and isolation in participants who presented a post-traumatic symptomatology. The results confirmed the negative role played by discrimination in influencing mental health [32], and are also in line with a recent study carried out in Southern Italy that showed the presence of high rates of violent episodes with racial discrimination experienced by immigrants and refugees [43]. As highlighted elsewhere [55, 57], loneliness, boredom, and isolation emerged as the basic condition of asylum seekers who experience a long-term identity and life suspension. Here, the high rates of loneliness, boredom, and isolation reported from the participants might also be seen in light of the “Security Decrees” which abolished most of the activities in which asylum seekers would have been occupied (i.e., Italian lessons). In this sense, the boredom could be interpreted as a “strategic boredom” [63], intended as a specific tool of strict control exercised on asylum seekers by the government to force them to be pointless and passive.

Furthermore, the comparison between the clinical and nonclinical group also showed that more individuals of Islamic religion were present in the PTSD group. This result confirmed a worsening in the mental health state if the asylum seekers experience religious alienation and exclusion in the host country [31], suggesting to deepen the mental health state of Muslim asylum seekers and the specific role played by religious discrimination on their well-being. Furthermore, in contrast with our assumption, no differences were found in the resilience factors.

To conclude, the authors believe the results could inform practitioners and professionals working in the reception field as well as orient the hospitality policies towards more person-centered and culturally informed actions and practices. First, the results shed light on the mental health state of a specific ethnic group and might help professionals to better understand the specific struggles, needs, and necessities of Nigerian male asylum seekers. In this sense, we believe mental health practitioners can strengthen their knowledge of Nigerian culture, incorporating this competence into interventions as core components of Nigerian asylum seekers’ identity and strategies to make sense of trauma. This might also be helpful in improving their professional practice, making it much more reflective, reducing the risk of self-discomfort, and allowing them to recognize the risks and resource factors for mental health and the way in which these might impact the taking charge of this population [18, 27] Second, even though some progress has been made in the last year within the Italian hospitality system to repair the damages produced by the “Security Decree,” many others need to be made to create an equal and right system of reception and hospitality able to take care of asylum seekers and refugees, rather than exacerbating their suffering and vulnerabilities. This is even more urgent in light of the COVID-19 pandemic whose impact on migrants and refugees is potentially greater as they are particularly vulnerable from a physical as well as psychological point of view.

The present study is not free from limitations. First of all, the number of participants resulted in a small sample for the analysis, even though several difficulties should be taken into account in accessing this specific population. Moreover, the CD-RISC measure used for the resilience levels showed lower values of Cronbach’s alpha in factors 2, 3, 4, and 5. Despite the threshold for good reliability (0.70), previous studies highlighted many pitfalls in using Cronbach’s alpha as a reliability index because it is strictly related to the targeted population [19, 60]. These values reveal the need to deepen the understanding of the resilience results. From the author’s point of view, CD-RISC may not have been effective in grasping the dimensions of resilience and the results suggested the need to achieve a higher accord around the conceptualization of this construct and, specifically, the development of adequate instruments to measure the resilience of refugees while taking into account the social, community, and cultural dimensions of resilience.

Broadly, even though the study presented the results of a quantitative clinical investigation, the authors would suggest to practitioners and clinicians to implement the integration of quantitative measures as well as qualitative ones, since the use of solely quantitative measures might be affected by several linguistic and cultural bias, offering a limited view of the complexity of the refugees’ mental health status. In this sense, the authors strongly encourage the use of qualitative methods with vulnerable and traumatized populations in order to enable the contact with the affective, emotional, and mind–body processes usually shutdown by trauma [14–17, 23, 24, 45, 48, 52]. Statistical analyses of the present study were chosen by taking into account the small sample size [20, 30]. Furthermore, due to this limitation, the bootstrap procedures were also used [8]. In conclusion, the results need to be replicated in other geographical areas and in areas with different neighborhood characteristics to determine their generalizability.
